# Accountability and Funding for State-Level School Physical Education and Recess Laws

**DOI:** 10.1016/j.amepre.2025.108017

**Published:** 2025-07-31

**Authors:** Hannah R. Thompson, Ursula Lochner-Bravo, Jonanne Talebloo, Jennie N. Davis, Jennifer Falbe

**Affiliations:** 1 Community Health Sciences, School of Public Health, University of California, Berkeley, Berkeley, California; 2 Institute for Global Nutrition, University of California, Davis, Davis, California; 3 Department of Human Ecology, University of California, Davis, Davis, California

## Abstract

School physical education and recess provide important opportunities to increase youth physical activity and socioemotional development. However, compliance with existing laws is low at the elementary level. Reported barriers to law implementation include limited accountability and funding. However, the number of state physical education and recess laws that stipulate accountability and/or funding remains unknown. This cross-sectional review of active U.S. state-level laws related to public elementary school physical education and recess was conducted between October and December 2024. The Classification of Laws Associated with School Students and the National Association of State Boards of Education databases, along with web searches, were used to identify laws. Laws were double coded for inclusion of accountability- and funding-related language. Forty-nine U.S. states (96.1%) had physical education laws, and 22 (43.1%) had recess law; 21 (41.2%) had both. Among states with physical education law, 12 (25.5%) and 1 (2.0%) included language on accountability and funding, respectively. Among states with recess laws, 5 (22.7%) included language related to accountability, and none contained funding-related language. State-level physical education and recess legislation is highly prevalent, but most laws do not contain accountability-related language; virtually none contain funding-related language, which may be inhibiting implementation and driving inequities in related health outcomes. Additional research examining the relationship between accountability and funding language in physical education and recess laws and the degree of implementation of the laws is necessary to help inform the development of stronger laws to support the provision of quality physical education and recess in schools nationally.

## INTRODUCTION

Regular physical activity provides a myriad of benefits for children, including improved cardiorespiratory health, stronger muscles and bones, enhanced academic performance and cognition, and improved sleep.^1^ As such, the HHS recommends that youth accrue 60 minutes of moderate-to-vigorous physical activity daily. The Institute of Medicine advises that this activity occurs during the school day, specifically.^2,3^ However, as of 2022, less than a quarter of U.S. school-aged children met the physical activity guideline.^4^

School physical education (PE) and recess provide important opportunities for children to achieve physical activity recommendations as well as confer positive academic, behavioral, emotional, and social impacts.^3,5–7^ Evidence demonstrates that states with strong laws are more likely to provide PE and recess to the benefit of physical and socioemotional student health.^8–10^ Although many states require PE through law, the average mandated time is significantly below the recommended 60 minutes of daily moderate-to-vigorous physical activity.^11^ In addition, when state PE and recess laws do exist, adherence is often low, particularly at the elementary level.^12,13^

Primary identified barriers to PE and recess law implementation include limited accountability and funding for these subjects.^14^ Although unfunded and unaccounted-for mandates continue to strain the education systems,^15^ the prevalence of such PE- and recess-specific laws remains unknown.

The purpose of this study is to quantify the number of state elementary PE and recess laws and the number that includes measures of accountability and funding. Findings can help inform the development of stronger laws to support the provision of quality PE and recess in schools nationally.

## METHODS

This cross-sectional study reviewed U.S. state-level laws related to public elementary school PE and recess that were available online from October to December 2024. Two researchers (UL-B and JT) first reviewed the CLASS (Classification of Laws Associated with School Students)^16^ database to identify states (*n*=51, including the District of Columbia) with codified PE and recess laws/policies and cross referenced this with the National Association of State Boards of Education State Policy database.^17^ Web searches identified additional legislation passed or modified since these 2 databases were last updated (2022).

Two researchers (UL-B and JT) downloaded all available laws, and each independently coded every law for language related to (1) accountability for the law (e.g., accountability, enforcement, reporting, monitoring, compliance) and (2) funding (e.g., funding, fund[s], money, appropriation[s]). All codes were compared, and discordant codes were reconciled by a third researcher (HRT). Descriptive statistics were used to determine the number of states with (1) active PE and recess laws and language related to (2) accountability and (3) funding for the law(s).

## RESULTS

As of December 2024, 49 U.S. states (96.1%) had an active law pertaining to elementary school PE, and 22 (43.1%) had an active law regarding recess; 21 (41.2%) had both (Table 1). Among state PE laws, 12 (25.5%) included language related to accountability. For example, Tennessee’s Education Code 49-6-102 says “Each LEA [local education agency] shall file an annual report with the commissioner of education verifying that the LEA has met the PE requirements of this section.” South Carolina’s Code of Laws 59-10-10 reads “Each school district shall establish and maintain a Coordinated School Health Advisory Council (CSHAC) to assess, plan, implement, and monitor district and school health policies and programs.” Table 2 contains exact language from each law.

Of all state PE laws, Illinois Compiled Statute 105 5/27-6 was the only one to contains language on evaluation and reporting requirements (led by the Department of Education), as follows:

“The Office of Teaching and Learning is authorized to oversee school implementation and compliance with this policy and, in doing so, shall…Establish a process to gather regular reporting and feedback from individual schools, community partners, students and parents on the implementation of the policy; Conduct periodic evaluations and upon request report on district-wide and individual schools’ compliance with the Policy to the Board.”

It also specified direct support to schools in ensuring compliance (technical assistance for PE implementation) and included language explicitly addressing potential inequities resulting from disparate implementation: “Shall…Establish a process for assessing the equity impact of this policy, including how the policy is implemented in relation to who is most impacted by inequity to determine targeted universalist support for schools.”

Among state recess laws, 5 (22.7%) included language related to accountability for the law. For example, Kentucky’s Instructional Policy 160.345 included “The Department of Education shall report to the Legislative Research Commission no later than November 1 of each year on how the schools are providing physical activity under this subsection and on the types of physical activity being provided.” California’s Senate Bill 291 (Education Code Section 49056) included “California Department of Education recommends if LEAs offer recess that they evaluate their bell schedules and instructional minute offerings to ensure compliance with the instructional time requirements and avoid any potential audit finding and penalty.”

Among state PE laws, only 1 (2.0%) included language related to funding for implementation. Oregon Revised Statute 329.504 included “The Oregon Department of Education will seek private and public funding for programs that link physical activity and academic achievement, along with providing school districts information about funding opportunities that are directly available to schools and school districts.” Among state recess laws, none (0%) included language related to funding for the law.

## DISCUSSION

This review of active (as of December 2024) U.S. state-level elementary school PE and recess laws shows that most laws do not contain specific language related to accountability, and virtually none include information on funding. Although PE and recess have been identified as important enough to mandate by law in most states (96% and 43%, respectively), these laws are only as effective as the resources available to ensure their successful implementation. Without the funding necessary to implement these subjects well and without incentives/penalties for noncompliance with state laws, unequal provision of both PE and recess is likely to continue to contribute to persistent health and academic disparities for students.^12,14,18,19^

Most laws that mentioned accountability did so in the context of placing the responsibility on schools and/or the school district to ensure their own compliance, using suggestive (e.g., should) versus directive (e.g., shall) language. For example, PE laws included language that said that local education agencies (LEAs) should self-report their own compliance with the law to the Department of Education or legislators (Georgia, New York, California, Tennessee); monitoring compliance is a district-by-district decision, and compliance can be assessed through posted schedules indicating school-wide physical activity times (Alaska); LEAs can assign a person or committee in each school to ensure compliance (South Carolina); schools should report a summary of physical activities students are exposed to in PE to parents annually (Oklahoma); schools that do not comply need to submit an action plan to the Department of Education to increase minutes (Washington, DC); or accountability for PE falls under general school accountability standards (Mississippi and Nevada).

Illinois’s state PE law was the only PE law to contain specific language on evaluation and reporting requirements. The law also specified technical assistance to ensure implementation of the law and addressed potential inequities resulting from unequal implementation. Prior meta-analysis evidence shows that students in states with strong PE laws spend an average additional 34 minutes participating in PE classes per week, demonstrating the clear value of strongly written legislation.^20^ Additional research on the relationship between the existence and strength of accountability and funding language in PE and recess laws and the degree of law implementation is necessary to ensure that future laws are as strong as possible.

Only 1 state (Oregon) had a PE law with funding-related language. Not a single state’s recess law mentioned appropriations or financial assistance for implementation. Too often, unfunded health-related mandates are passed but place the onus on already under-resourced schools to improve student outcomes without additional support.^15^ Prior research on PE in California demonstrates that unfunded legislation requiring schools to do more, without providing the direct financial or structural support schools need, can result in low compliance in already overburdened, under-resourced schools, worsening existing inequities.^14^ By contrast, evidence from the New York City Department of Education demonstrates the positive impact of funding and supporting the implementation of PE legislation on both school culture and student health.^21,22^

This work is not without limitations. This study focused on active U.S. state-level legislation, but county-, city-, or district-level laws and policy from other countries can inform and innovate domestic legislation. In addition, draft/in-process legislation was not included. Although several state laws contain language related to staffing/teacher requirements, which can indicate support for PE and recess, staffing was not considered to be a clear indicator of accountability or funding unless it was accompanied by language related to tracking/reporting staffing or financial support for the position(s). Finally, the legislation search was not updated past December 2024.

## CONCLUSIONS

State-level PE and recess legislation is highly prevalent, but compliance with these laws is often low. This study provides the first known account of the inclusion of accountability- and funding-related measures in state-level PE and recess legislation, demonstrating that most laws do not contain specific language related to accountability, and virtually none contain language on funding. Additional research examining the relationship between accountability and funding language in PE and recess laws and the degree of implementation of the laws is necessary to understand whether continuing to pursue universal school-based mandates is worthwhile and/or whether more targeted (and funded) policies are warranted to improve implementation of and compliance with such laws.

## Figures and Tables

**Table 1.** Prevalence of U.S. Public Elementary School Laws With Elementary School PE and Recess Laws

State (*n*=51)	State has PE law	PE law includes language related to accountability	PE law includes language related to funding	State has recess law	Recess law includes language related to accountability	Recess law includes language related to funding
Alabama	Yes	No	No	No	N/A	N/A
Alaska	Yes	Yes	No	Yes	No	No
Arizona	Yes	No	No	Yes	No	No
Arkansas	Yes	No	No	Yes	No	No
California	Yes	Yes	No	Yes	Yes	No
Colorado	No	N/A	N/A	Yes	Yes	No
Connecticut	Yes	No	No	Yes	No	No
Delaware	Yes	No	No	No	N/A	N/A
District of Columbia	Yes	Yes	No	Yes	No	No
Florida	Yes	No	No	Yes	No	No
Georgia	Yes	Yes	No	Yes	No	No
Hawaii	No	N/A	N/A	No	N/A	N/A
Idaho	Yes	No	No	No	N/A	N/A
Illinois	Yes	Yes	No	Yes	No	No
Indiana	Yes	No	No	Yes	No	No
Iowa	Yes	No	No	No	N/A	N/A
Kansas	Yes	No	No	No	N/A	N/A
Kentucky	Yes	No	No	Yes	Yes	No
Louisiana	Yes	No	No	Yes	No	No
Maine	Yes	No	No	No	N/A	N/A
Maryland	Yes	No	No	No	N/A	N/A
Massachusetts	Yes	No	No	No	N/A	N/A
Michigan	Yes	No	No	No	N/A	N/A
Minnesota	Yes	No	No	No	N/A	N/A
Mississippi	Yes	Yes	No	No	N/A	N/A
Missouri	Yes	No	No	Yes	No	No
Montana	Yes	No	No	No	N/A	N/A
Nebraska	Yes	No	No	No	N/A	N/A
Nevada	Yes	Yes	No	No	N/A	N/A
New Hampshire	Yes	No	No	Yes	No	No
New Jersey	Yes	No	No	Yes	No	No
New Mexico	Yes	No	No	No	N/A	N/A
New York	Yes	Yes	No	No	N/A	N/A
North Carolina	Yes	No	No	No	N/A	N/A
North Dakota	Yes	No	No	No	N/A	N/A
Ohio	Yes	No	No	No	N/A	N/A
Oklahoma	Yes	Yes	No	No	N/A	N/A
Oregon	Yes	Yes	Yes	No	N/A	N/A
Pennsylvania	Yes	No	Yes	No	N/A	N/A
Rhode Island	Yes	No	No	Yes	Yes	No
South Carolina	Yes	Yes	No	No	N/A	N/A
South Dakota	Yes	No	No	No	N/A	N/A
Tennessee	Yes	Yes	No	Yes	Yes	No
Texas	Yes	No	No	Yes	No	No
Utah	Yes	No	No	No	N/A	N/A
Vermont	Yes	No	No	Yes	No	No
Virginia	Yes	No	No	No	N/A	N/A
Washington	Yes	No	No	Yes	No	No
West Virginia	Yes	No	No	Yes	No	No
Wisconsin	Yes	No	No	No	N/A	N/A
Wyoming	Yes	No	No	No	N/A	N/A
All states, *n* (%)	49 (96.1)	12 (24.5)	1 (2.0)	22 (43.1)	5 (22.7)	0 (0.0)

N/A, not applicable; PE, physical education.

**Table 2.** Exact Language Related to Accountability and Funding Found in Public Elementary School Laws With Elementary School PE and Recess Laws

State (*n*=51)	PE law exact language related to accountability	PE law exact language related to funding	Recess law exact language related to accountability	Recess law exact language related to funding
Alabama	None	None	No recess law	No recess law
Alaska	Who is going to monitor compliance? Designation of this role is a district by district decision. Compliance can be assessed through posted schedules indicating school wide physical activity times like recess or movement breaks, as well as sample lesson plans indicating classroom-based physical activity. Reporting on implementation of the Mandatory Physical Activity in Schools law should be incorporated in the district’s next required review of the Student Nutrition and Physical Activity policy (aka Wellness Policy).(Link to law)	None	None	None
Arizona	None	None	None	None
Arkansas	None	None	None	None
California	(1) Each school district selected by the Superintendent of Public Instruction pursuant to paragraph (2) shall report to the Superintendent of Public Instruction in the Coordinated Compliance Review as to the extent of its compliance with subdivision (g) of Section 51210 for grades 1 to 6, inclusive, during that school year. (2) The Superintendent of Public Instruction shall select not less than 10 percent of the school districts of the state to report compliance with the provisions set forth in paragraph (1). The school districts selected shall provide a random and accurate sampling of the state as a whole. (Link to law)	None	California Department of Education recommends if LEAs offer recess that they evaluate their bell schedules and instructional minute offerings to ensure compliance with the instructional time requirements and avoid any potential audit finding and penalty. (Link to law)	None
Colorado	No PE law	No PE law	Each school district board of education may require the person or committee in each school designated to ensure that the school complies with the local wellness policy, as described in section 22-32-136, or the school district accountability committee and school accountability committees created pursuant to article 11 of this title to review and advise the school district or an individual school regarding the school district’s or the individual school’s physical activity policy and compliance with this section (Link to law)	None
Connecticut	None	None	None	None
Delaware	None	None	No recess law	No recess law
District of Columbia	A school that provides less than an average of 90 minutes per week of physical education in a school year for students in grades kindergarten through 5 shall submit an action plan to the Office of the State Superintendent of Education detailing efforts it will take to increase physical education before the beginning of the next school year and shall work with the Office of the State Superintendent of Education to increase the amount of time provided for physical education each week. (Link to law)	None	None	None
Florida	None	None	None	None
Georgia	The State Board of Education shall submit an annual report to the Governor, beginning October 1, 2012, and annually thereafter. Such report shall include the compliance status of each local school system and each school with applicable State Board of Education rules and regulations. (Link to law)	None	None	None
Hawaii	No PE law	No PE law	No recess law	No recess law
Idaho	None	None	No recess law	No recess law
Illinois	The Office of Teaching and Learning is authorized to oversee school implementation and compliance with this policy and, in doing so, shall: Provide technical assistance and support to schools with implementation of the policy and improve programming functions; Ensure schools are offered support services through various Central Office departments and Network offices; Establish a credential process for outside partners and community agencies to support schools; Establish a process to gather regular reporting and feedback from individual schools, community partners, students and parents on the implementation of the policy; Conduct periodic evaluations and upon request report on district-wide and individual schools’ compliance with the Policy to the Board; Monitor individual student exemption requests granted by high schools; Establish a process for assessing the equity impact of this policy, including how the policy is implemented in relation to who is most impacted by inequity to determine targeted universalist support for schools. (Link to law)	None	None	None
Indiana	None	None	None	None
Iowa	None	None	No recess law	No recess law
Kansas	None	None	No recess law	No recess law
Kentucky	None	None	The department of education shall report to the Legislative Research Commission no later than November 1 of each year on how the schools are providing physical activity under this subsection and on the types of physical activity being provided (Link to law)	None
Louisiana	None	None	None	None
Maine	None	None	No recess law	No recess law
Maryland	None	None	No recess law	No recess law
Massachusetts	None	None	No recess law	No recess law
Michigan	None	None	No recess law	No recess law
Minnesota	None	None	No recess law	No recess law
Mississippi	In accordance with Miss. Code Ann. §37-13-134 (Mississippi Healthy Students Act) and the Mississippi Public School Accountability Standards, the State Board of Education has adopted the following rules and regulations to support the implementation of quality activity based and health education programs (Link to law)	None	No recess law	No recess law
Missouri	None	None	None	None
Montana	None	None	No recess law	No recess law
Nebraska	None	None	No recess law	No recess law
Nevada	The board of trustees of each school district shall conduct a periodic review of the courses of study offered in the public schools of the school district to determine whether the courses of study comply with the standards of content and performance established by the Council pursuant to NRS 389.520 and if revision of the courses of study is necessary to ensure compliance. (Link to law)	None	No recess law	No recess law
New Hampshire	None	None	None	None
New Jersey	None	None	None	None
New Mexico	None	None	No recess law	No recess law
New York	Periodic reports regarding the status and progress of equivalent programs which have been approved by the commissioner shall be filed with the Division of Physical Education, Fitness, Health, Nutrition and Safety Services as requested. (Link to law)	None	No recess law	No recess law
North Carolina	None	None	No recess law	No recess law
North Dakota	None	None	No recess law	No recess law
Ohio	None	None	No recess law	No recess law
Oklahoma	School districts shall provide to parents or guardians of students a physical activity report. The report shall be provided to parents and guardians at least annually and shall include: 1. A summary on how physical activity is being incorporated into the school day; 2. A summary of the types of physical activities the students are exposed to in the physical education programs; 3. Suggestions on monitoring the physical activity progress of a child and how to encourage regular participation in physical activity; and; 4. Information on the benefits of physical education and physical activity. (Link to law)	None	No recess law	No recess law
Oregon	A school district that does not comply with the requirements of this section is considered to be nonstandard under ORS 327.103 (Link to law)	The Oregon Department of Education will seek private and public funding for programs that link physical activity and academic achievement, along with providing school districts information about funding opportunities that are directly available to schools and school districts for programs that link physical activity and academic achievement. ORS 329.504 (Link to law)	No recess law	No recess law
Pennsylvania	None	None	No recess law	No recess law
Rhode Island	None	None	The Health and Wellness Sub-Committee and None Superintendent, working in conjunction with school principals, shall assess the District’s policies and implementation on an annual basis, and shall forward recommendations for improvement based on the assessment data and any new federal/state regulation and/or national best practice to the full school committee. A report will be made available to the school community and the public and will include the following: The extent to which schools are in compliance with the wellness policy Obstacles to meeting compliance of the elements of the wellness policy Progress made in attaining the goals of the wellness policy How the wellness policy compares to model wellness policies (Link to law)	None
South Carolina	Each school district shall establish and maintain a Coordinated None School Health Advisory Council (CSHAC) to assess, plan, implement, and monitor district and school health policies and programs, including the development of a district wellness policy to begin implementation in the 2006-07 school year. (Link to law)	None	No recess law	No recess law
South Dakota	None	None	No recess law	No recess law
Tennessee	Each LEA shall file an annual report with the commissioner of education verifying that the LEA has met the physical education requirements of this section. (Link to law)	None	The office of coordinated school health in the department of education shall provide an annual report by October 1, to the education committees of the house of representatives and the education committee of the senate on the implementation of subsection (a). The report shall contain at least the following information: (1) The percentage of public schools that integrate the required physical activity into the instructional school day in compliance with subsection (a); (2) The types of physical activities that are used to meet the physical activity requirement; (3) Any barriers that have limited full compliance with the physical activity requirement; (4) Innovative methods that schools use to comply with the physical activity requirement; (5) The ranking of Tennessee schools in providing physical activity and physical education as compared to other states; (6) Relevant data or studies that link physical activity or physical education to academic performance in students; (7) Relevant data or studies showing whether increased physical activity or physical education lead to better health outcomes; (8) The annual percentage of increase or decrease in compliance with the physical activity requirement in school districts with average daily membership of twenty-five thousand (25,000) or more students; and (9) An overall summary and a set of recommendations to promote active living in the youth of this state, including, but not limited to, suggestions for increasing compliance with the physical activity requirement that can be implemented with minimal cost. (Link to law)	None
Texas	None	None	None	None
Utah	None	None	No recess law	No recess law
Vermont	None	None	None	None
Virginia	None	None	No recess law	No recess law
Washington	None	None	None	None
West Virginia	None	None	None	None
Wisconsin	None	None	No recess law	No recess law
Wyoming	None	None	No recess law	No recess law

LEA, local education agency; ORS, Oregon Revised Statutes; PE, physical education.
